# *In silico* modeling of directed differentiation of induced pluripotent stem cells to definitive endoderm

**DOI:** 10.1371/journal.pcbi.1013407

**Published:** 2025-08-21

**Authors:** Amirmahdi Mostofinejad, David A. Romero, Dana Brinson, Thomas K. Waddell, Golnaz Karoubi, Cristina H. Amon

**Affiliations:** 1 Department of Mechanical and Industrial Engineering, University of Toronto, Toronto, Ontario, Canada; 2 Institute of Biomedical Engineering, University of Toronto, Toronto, Ontario, Canada; 3 Latner Thoracic Surgery Research Laboratories, Toronto General Hospital Research Institute, University Health Network, Toronto, Ontario, Canada; 4 Institute of Medical Sciences, University of Toronto, Toronto, Ontario, Canada; 5 Department of Laboratory Medicine and Pathobiology, University of Toronto, Toronto, Ontario, Canada; University of Melbourne, AUSTRALIA

## Abstract

Differentiation of embryonic stem cells and induced pluripotent stem cells (iPSCs) into endoderm derivatives, including thyroid, thymus, lungs, liver, and pancreas, has broad implications for disease modeling and therapy. We utilize and expand a model development approach previously outlined by the authors to construct a model for the directed differentiation of iPSCs into definitive endoderm (DE). Assuming discrete intermediate stages in the differentiation process with a homogeneous population in each stage, three lineage models with two, three, and four populations and three growth models are constructed. Additionally, three models for error distribution are defined, resulting in a total of 27 models. Experimental data obtained *in vitro* are used for model calibration, model selection, and final validation. Model selection suggests that no transitory state during differentiation expresses the DE biomarkers CD117 and CD184, a finding corroborated by existing literature. Additionally, space-limited growth models, such as logistic and Gompertz growth, outperform exponential growth. Validation of the inferred model with leave-out data results in prediction errors of 26.4%. Using the inferred model, it is predicted that the optimal differentiation period is between 1.9 and 2.4 days, plating populations closer to 300 000 cells per well result in the highest yield efficiency, and that iPSC differentiation outpaces the DE proliferation as the main driver of the population dynamics. We also demonstrate that the model can predict the effect of growth modulators on cell population dynamics. Our model serves as a valuable tool for optimizing differentiation protocols, providing insights into developmental biology.

## Author summary

The directed differentiation of induced pluripotent stem cells (iPSCs) into definitive endoderm (DE) represents the first step in deriving many endoderm-derived tissues, such as lung, liver, and pancreas cells. Here, we report a mathematical model of iPSC differentiation into DE, thus providing a tool for optimizing directed differentiation protocols. Biologically informed models are developed using experimental data to capture population dynamics, including proliferation, differentiation, and death. The optimal lineage model and growth model are identified for the differentiation process by performing model selection and validation. Model validation is performed by calculating the normalized root-mean-square error on leave-out data to be 26.4%, which is below the threshold of 30%. Using the validated model, we examined questions related to the optimization of DE induction. Model predictions suggest that the optimal differentiation period is approximately 2 days and that the optimal plating population is 300,000 cells per well. We also conducted an *in silico* investigation into the effect of supplementation of culture medium with growth modulators on the yield of DEs. We found that this may lead to an increase in DE yield of up to 39% at lower plating populations. These studies showcase the value of mathematical modeling in optimizing cell culture protocols by reducing the need for extensive experimentation in the laboratory. The model also provides insight into the differentiation process, aiding developmental biology and regenerative medicine research.

## Introduction

Embryonic (ESCs) and induced pluripotent stem cells (iPSCs) can be differentiated into various types of cells, including ectoderm, mesoderm, and endoderm derivatives [1]. The latter include the thyroid, thymus, lungs, liver, pancreas, and epithelial lining of the respiratory and digestive systems [2–5]. The directed differentiation of iPSCs to endoderm derivatives begins with the induction of definitive endoderm (DE) [6,7], showing the importance of this stage in disease modeling, drug discovery, personalized medicine, and engineered patient-specific tissues [8].

The DE induction protocol optimization methods have primarily relied on *in vitro* and *ex vivo* experiments to understand the activated pathways and the required nutrients and growth factors for cellular patterning [9]. State-of-the-art studies had limited attention to utilizing *in silico* modeling needed for accelerated discovery by reducing time, cost, and variance and improving cell yields [10]. Consequently, mathematical models to predict the iPSC differentiation into DE can potentially augment future experimental design and optimization processes while offering a deeper insight into the complex dynamics of the population system [11].

Several mathematical models have been employed to investigate stem cell differentiation. Due to the time dependency of the differentiation process, one approach for mathematical modeling is the use of differential equations [12]. Initial modeling attempts focused on the theoretical aspect, without experimental data used to calibrate or validate them, primarily aimed to reproduce qualitative behaviors [13–16]. These models facilitate a theoretical understanding of the fundamental principles governing stem cell differentiation, including the balance between self-renewal and differentiation, the role of feedback mechanisms, and the impact of stochasticity on cell fate decisions. They also provide a framework for exploring how various parameters influence system behavior, enabling the generation of hypotheses about underlying biological processes [17].

A further step is to incorporate experimental data to calibrate the models, thereby enhancing their applicability to real-world systems [18,19]. These calibrated models have significantly enhanced our understanding of stem cell differentiation by providing quantitative insights into the dynamics of cell populations. However, in most cases, the proposed models have not been analyzed for parameter uniqueness or identifiability, a prerequisite for model and parameter interpretability [18,20].

We utilize a previously reported model development approach, with a focus on modeling multicellular populations undergoing differentiation [21]. Herein, models are enhanced to include a structural component incorporating the expected values and an error component including the standard deviations. This novel and enhanced model will be a tool for furthering our understanding of the differentiation process of iPSCs, enabling optimization for numerous applications.

Here, we present a mathematical model to predict the population dynamics of directed iPSC differentiation to DE. Model inference begins with multiple biology-informed model proposals that consider varying cellular differentiation lineages and cell growth models. Candidate models consider various states through which stem cells transition to a differentiated state. We then infer these models using *in vitro* observations from designed experiments to maximize each model’s prediction accuracy. We select the most suitable model by subjecting these models to rigorous selection processes and ensuring model identifiability using mathematical tests. Finally, we validate the model by examining its prediction errors over a subset of the data not used for model inference and selection.

The following sections detail the experimental setup, the modeling assumptions, and the resulting candidate models and loss measures. In the Results section, the model calibration, selection, and validation are described, parameter uniqueness is established [20], and parameters are ranked by their impact on the cell populations [22]. The Applications section includes two *in silico* experiments that are run using the inferred model to optimize the differentiation timespan and the yield per input cell while exploring the effect of hypothetical added Rho-associated protein kinase inhibitor (ROCKi) to the culture media. Finally, the Discussion section illustrates how the model facilitates a deeper understanding of the dynamics of the directed differentiation of iPSCs to DE.

## Methods

### Experimental setup

#### Cell Culture.

**Fig 1:**
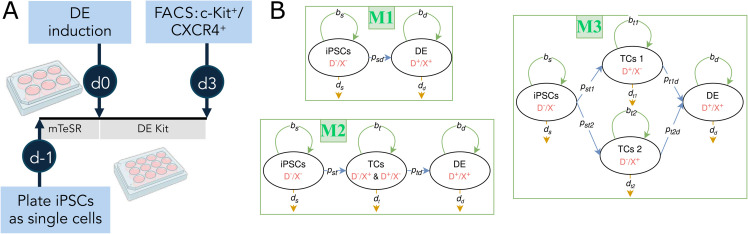
Experimental protocol and the lineage models. (A) Experimental protocol. Typical differentiation timeline for DE induction of iPSCs. Created with Biorender. (B) Lineage models. TCs, iPSCs, and DE are transition cells, induced pluripotent stem cells, and definitive endoderm, respectively. Also, mTeSR, DE Kit, and FACS represent mTeSR-1 iPSC maintenance medium, STEMdiff Definitive Endoderm Kit, and fluorescence-activated cell sorting, respectively. Biomarker expressions are shown as D^ + ^ for CD117+, D^−^ for CD117-, X^ + ^ for CD184+, and X^−^ for CD184-. Green, blue, and orange lines correspond to proliferation, differentiation, and death rates, respectively.

iPSCs are maintained as colonies on Matrigel (Corning, cat. no. 354277)-coated 6-well plates. Prior to beginning differentiation, cells are first passaged as single cells onto Matrigel-coated 12-well plates and cultured in the iPSC maintenance medium (mTeSR-1, StemCell Technologies, cat. no. 85850) for 24 hours with Y-27632 (10 μM, StemCell Technologies, cat no. 72304). Then, the cells are cultured with STEMdiff Definitive Endoderm Kit (StemCell Technologies, cat. no. 05110) for 120 hours, longer than the protocol’s recommended 72 to 96 hours, to observe the effect of elongating this step (Fig 1A).

#### Measurements.

Each day, media samples of 160 μL were taken from all wells and measured glucose and lactate concentrations using RAPIDPoint 500 Blood Gas Systems (Siemens Healthcare Limited, Canada). Then, a well was harvested daily.

#### Harvesting wells.

The well to be harvested was first washed with DMEM/F12 (Thermo Fisher, cat. no. 11330-057). Cells were dissociated into single cells by incubating with GCDR (StemCell Technologies, cat. no. 07174) for 4 minutes. The first DMEM/F12 wash solution was added to the GCDR single-cell solution to ensure accurate counts of the live and dead cells. A 20 μL sample of the total cell solution is used for counting using a hemocytometer, and the rest were analyzed using flow cytometry.

#### Flow Cytometry.

The efficiency of DE induction was determined by assessing the co-expression of c-KIT-APC (1 μL per 1 million cells; eBioscience, cat. no. 5015059) and CXCR4-PE-Cy7 (1 μL per 1 million cells; Becton Dickinson cat. no. 560669) surface markers (N=3 BU3 NGST hiPSC line, kindly provided by CReM at Boston University), and stained with 4’, 6-diamidino-2-phenylindole (DAPI $1\;\mu {g\;mL}^{-1}$; Millipore Sigma, D9542-1MG). Samples were acquired on a BD LSRFortessa Cell Analyzer (Becton Dickinson) and analyzed using FlowJo software (Becton Dickinson).

### Model proposal

#### Structural models.

The base assumption in formalizing the structural models is that there are finite stages in the differentiation of the iPSCs to DE. Cells transfer between the stages according to a predetermined graph (Fig 1B) with mechanisms such as proliferation, death, and differentiation [13]. Also, cell populations in each compartment are assumed to be spatially homogeneous due to the initial spatial homogeneity and the homogeneity of nutrient and waste concentrations in the monolayer culture system.

As seen in Fig 1B, three potential iPSC differentiation paths are investigated. The two principal live cell populations that define these routes are the undifferentiated iPSCs and DE, denoted by *n*_*s*_ and *n*_*d*_, respectively. In this study, we use the co-expression of markers CD184 (CXCR4) and CD117 (c-KIT), which are commonly used markers of DE [9,23–25]. In addition, transitory states can be defined as cells that express CD117 but not CD184; we refer to them as transition cells 1, *n*_*t*1_. The opposing marker profile, CD184+/CD117-, indicates another transitional population referred to as transition cells 2, *n*_*t*2_. By aggregating these two transitional populations, we define a combined transition population, *n*_*t*_. Moreover, $n_{\tilde{q}}$ represents the total dead cell population.

The model also contains the per capita death, *d*_*j*_, and proliferation rates, *b*_*j*_, for each live population, *n*_*j*_. It also includes the per capita differentiation rate $p_{jj^{\prime }}$ from population *n*_*j*_ to $n_{j^{\prime }}$. Crucially, it is assumed that these cellular processes are only impacted by the size of the particular cell populations involved and are not dependent on biochemical concentrations, which is compatible with running all the experiments in the condition specified by STEMdiff Definitive Endoderm Kit protocol. The implicit assumption is that the environment provides required nutrients and growth factors at the necessary concentrations and removes sufficient waste so that the cells can grow independent of significant changes in the culture environment. Also, the death rate is assumed to be a constant $\delta_{j}$. The rates are defined as,

$$b_{j}\left(𝐍(t),𝐂(t)\right)=b_{j}(n_{j}),\;p_{jj^{\prime }}\left(𝐍(t),𝐂(t)\right)=p_{jj^{\prime }}(n_{j}),\;d_{j}\left(𝐍(t),𝐂(t)\right)=\delta_{j},$$ (1)

where all the populations and the concentrations are contained in two vectors, 𝐍(t) and 𝐂(t), respectively.

#### Lineage model I.

Using Fig 1B, model M1 is defined as,

$$\frac{\text{d}n_{s}}{\text{d}t}=b_{s}\left(n_{s}\right)n_{s}-\delta_{s}n_{s}-p_{sd}\left(n_{s}\right)n_{s},\;\frac{\text{d}n_{d}}{\text{d}t}=b_{d}\left(n_{d}\right)n_{d}-\delta_{d}n_{d}+p_{sd}\left(n_{s}\right)n_{s},\;\frac{\text{d}n_{\tilde{q}}}{\text{d}t}=\delta_{s}n_{s}+\delta_{d}n_{d}.$$ (2)

This model has two live cell populations and the total dead cell population. Per capita proliferation and differentiation are defined as,

$$b_{s}\left(n_{s}\right)=\beta_{s}f\left(n_{s}\right),b_{d}\left(n_{d}\right)=\beta_{d}f\left(n_{d}\right),\;p_{sd}\left(n_{s}\right)=2\left(1-{𝗉}_{sd}\right)b_{s}\left(n_{s}\right).$$ (3)

As seen above, iPSCs proliferate at a rate $\beta_{s}f(n)$, resulting in two daughter stem cells with the probability of ${𝗉}_{sd}$ and generation of two differentiated cells with the probability of $\left(1-{𝗉}_{sd}\right)$ through symmetric division [13,16]. Also, $\beta_{s}$ and $\beta_{d}$ are the maximum proliferation rates for iPSCs and DE, respectively. Per capita growth rate, *f*(*n*), is defined later.

#### Lineage model II.

Considering a transitory cell state in which only either of the biomarkers is found in the cells, model M2 is defined with a structure similar to M1 with an additional state in the middle (Fig 1B). The mathematical form of the model is,

$$\frac{\text{d}n_{s}}{\text{d}t}=b_{s}\left(n_{s}\right)n_{s}-\delta_{s}n_{s}-p_{st}\left(n_{s}\right)n_{s}\;\frac{\text{d}n_{t}}{\text{d}t}=b_{t}\left(n_{t}\right)n_{t}-\delta_{t}n_{t}+p_{st}\left(n_{s}\right)n_{s}-p_{td}\left(n_{t}\right)n_{t}\;\frac{\text{d}n_{d}}{\text{d}t}=b_{d}\left(n_{d}\right)n_{d}-\delta_{d}n_{d}+p_{td}\left(n_{t}\right)n_{s}\;\frac{\text{d}n_{\tilde{q}}}{\text{d}t}=\delta_{s}n_{s}+\delta_{t}n_{t}+\delta_{d}n_{d}$$ (4)

The rates follow the same structure seen in M1 as,

$$b_{s}\left(n_{s}\right)=\beta_{s}f\left(n_{s}\right),b_{t}\left(n_{t}\right)=\beta_{t}f\left(n_{t}\right),\;p_{st}\left(n_{s}\right)=2\left(1-{𝗉}_{st}\right)b_{s}\left(n_{s}\right),b_{d}\left(n_{d}\right)=\beta_{d}f\left(n_{d}\right),\;p_{td}\left(n_{t}\right)=2\left(1-{𝗉}_{td}\right)b_{t}\left(n_{t}\right).\;$$ (5)

Note that ${𝗉}_{td}$ and ${𝗉}_{st}$ are defined similarly to ${𝗉}_{sd}$ in lineage model I.

#### Lineage model III.

The most descriptive model is M3, in which iPSCs can differentiate into two different cell populations, *n*_*t*1_ and *n*_*t*2_, either way, they can only differentiate into DE (Fig 1B). The process is mathematically defined as,

$$\frac{\text{d}n_{s}}{\text{d}t}=b_{s}\left(n_{s}\right)n_{s}-\delta_{s}n_{s}-p_{st1}\left(n_{s}\right)n_{s}-p_{st2}\left(n_{s}\right)n_{s}\;\frac{\text{d}n_{t1}}{\text{d}t}=b_{t1}\left(n_{t1}\right)n_{t1}-\delta_{t1}n_{t1}+p_{st1}\left(n_{s}\right)n_{s}-p_{t1d}\left(n_{t1}\right)n_{t1}\;\frac{\text{d}n_{t2}}{\text{d}t}=b_{t2}\left(n_{t2}\right)n_{t2}-\delta_{t2}n_{t2}+p_{st2}\left(n_{s}\right)n_{s}-p_{t2d}\left(n_{t2}\right)n_{t2}\;\frac{\text{d}n_{d}}{\text{d}t}=b_{d}\left(n_{d}\right)n_{d}-\delta_{d}n_{d}+p_{t1d}\left(n_{t1}\right)n_{t1}+p_{t2d}\left(n_{t2}\right)n_{t2}\;\frac{\text{d}n_{\tilde{q}}}{\text{d}t}=\delta_{s}n_{s}+\delta_{t1}n_{t1}+\delta_{t2}n_{t2}+\delta_{d}n_{d}$$ (6)

The rates follow a similar structure as the M1 and M2 with minor changes as,

$$b_{s}\left(n_{s}\right)=\beta_{s}f\left(n_{s}\right),\;\;b_{t1}\left(n_{t1}\right)=\beta_{t1}f\left(n_{t1}\right),\;b_{d}\left(n_{d}\right)=\beta_{d}f\left(n_{d}\right),\;\;b_{t2}\left(n_{t2}\right)=\beta_{t2}f\left(n_{t2}\right),\;p_{st1}\left(n_{s}\right)=2\left(1-{𝗉}_{st}\right){𝗉}_{t1}b_{s}\left(n_{s}\right),\;p_{st2}\left(n_{s}\right)=2\left(1-{𝗉}_{st}\right)\left(1-{𝗉}_{t1}\right)b_{s}\left(n_{s}\right),\;p_{t1d}\left(n_{t}\right)=2\left(1-{𝗉}_{t1d}\right)b_{t1}\left(n_{t1}\right),\;p_{t2d}\left(n_{t}\right)=2\left(1-{𝗉}_{t2d}\right)b_{t2}\left(n_{t2}\right).$$ (7)

Here, ${𝗉}_{st}$ is the probability of the daughter cells undergoing self-renewal, and ${𝗉}_{t1}$ is the probability of the differentiating cells becoming transitory cells 1. Also, ${𝗉}_{t1d}$ and ${𝗉}_{t2d}$ are defined similarly to ${𝗉}_{sd}$ in lineage model I.

#### Growth models.

The next component of the model to be defined is the per capita growth rate, *f*(*n*), and there are multiple ways to define it as,

$$f(n)=1\text{Exponential,}\;f(n)=\left(1-\frac{n}{n_{\max}}\right)\text{Logistic,}\;f(n)=\log\left(\frac{n_{\max}}{n}\right)\text{Gompertz.}$$ (8)

In these equations, $n_{\max}$ is the maximum population caused by limited space, nutrients, and waste removal. Lineage and growth models comprise the structural model denoted by **g**.

#### Error models.

The experimental observations, or observables **z**, are expressed as the summation of structural model, **g**, and the error term, ϵη as follows,

𝐳(𝐘,Θ,ξ,𝐮)=𝐠(𝐘,Θ,𝐮)+ϵ(𝐘,Θ,ξ,𝐮)η, (9)

In this equation, Θ, **Y**, **u**, ξ, are vectors containing model parameters, state variables, external stimuli, and error parameters, respectively. Also, η is the vector of independent random variables drawn from a Gaussian distribution with zero mean and unit standard deviation, defined as,

η∼𝒩(0,𝐈), (10)

here, **0** is a zero vector and **I** is the identity matrix [26]. Eq 9 also introduced the function ϵ which is the error model, with three different formulations proposed here as,

$$\epsilon_{j}=a\text{Additive,}\;\epsilon_{j}=b\cdot g_{j}(𝐘,\Theta ,𝐮)\text{Proportional,}\;\epsilon_{j}=a+b\cdot g_{j}(𝐘,\Theta ,𝐮)\text{Combined,}$$ (11)

where different *j*s refer to different observables [18,26]. Note that Eq 9 expresses the observations in terms of their mean value, 𝔼, and standard deviation, sd, shown as,

𝔼(𝐳∣Θ)=𝐠(𝐘,Θ,𝐮),sd(𝐳∣Θ,ξ)=ϵ(𝐘,Θ,ξ,𝐮). (12)

Finally, note that in the general case, the dimensions of **z** are a subset of **Y**. However, in this paper, since values for all the state variables are collected in the experiments, **z** and **Y** have the same dimensions.

Error models are necessary for experiments with few replicates, causing unreliable data standard deviations. Error models can also quantify the dependence of the standard deviation on expected values [26]. Incorporating error models requires a general loss function, which enables the simultaneous inference of the parameters of both structural and error models.

### Objective function definition

Estimating model parameters from data can be formulated as an optimization problem, where model parameters (structural parameters, Θ, and error parameters, ξ) are estimated that maximize an objective, here the likelihood function. The likelihood function, ℒ, for independent observations, *z*_*i*_, is defined as the product of their probability density functions, *p*, as,

$$ℒ(\Theta ,\xi ;𝐳)=\prod_{i=1}^{n}p(\Theta ,\xi ;z_{i}).$$ (13)

Under the assumption that the normalized residuals η=(𝐳−𝐠)/ϵ are independent and identically distributed, η∼N(0,I), Eq 13 is equivalent to minimizing the negative log-likelihood [27]. So, the problem becomes finding the inferred structural $\Theta^{*}$, and error parameters $\xi^{*}$, defined by the minimization problem below,

$$(\Theta^{*},\xi^{*})=\arg\min_{\Theta ,\xi }(-2\ell (\Theta ,\xi ))\;=\arg\min_{\Theta ,\xi }\sum_{g_{j}\in 𝐠}\sum_{Z_{j_{k}}\in {𝒮}_{T}}w_{j}\left(\Theta ,\xi ,t_{k}\right)(Z_{j_{k}}-g_{j}\left(\Theta ,t_{k}\right){)}^{2}\;+\sum_{g_{j}\in 𝐠}\sum_{Z_{j_{k}}\in {𝒮}_{T}}2\ln(\epsilon_{j}\left(\Theta ,\xi ,t_{k}\right)).$$ (14)

Here, the weights are $w_{j}(\Theta ,\xi ,t_{k})=\sigma_{j}^{-2}(\Theta ,\xi ,t_{k})=\epsilon_{j}^{-2}(\Theta ,\xi ,t_{k})$. Also, ℓ, *g*_*j*_, $Z_{j_{k}}$, ln, and ${𝒮}_{T}$ are the log-likelihood objective function, model predictions, experimental measurements, natural logarithm function, and training dataset, respectively [28,29]. When ϵ takes the additive (constant variance) form in Eq 11, the second term is constant, and the objective simplifies to weighted nonlinear least squares plus a constant [20]. The observables for Eq 14 are the state variables in equations 2, 4, 6 corresponding to each of the models.

### Structural identifiability analysis

Structural identifiability is equivalent to the injectivity of the map from parameters to observations. In simpler terms, identifiability of a model is the existence of a unique set of parameters corresponding to a set of infinite noiseless observations [30–32]. Mathematically, if Θ and Φ are two valid sets of structural model parameters, then,

𝐠(𝐘,Θ,𝐮)=𝐠(𝐘,Φ,𝐮)⟹Θ=Φ. (15)

Structural identifiability analysis is independent of the data and only focuses on the relationship between state variables and observables, i.e., the model equations and the measured states. So, structural unidentifiability of a model results in it being discarded (or reparameterized) prior to calibration [27,33]. The analysis often involves applying differential algebra techniques to derive input-output equations from the model. From these, a symbolic identifiability matrix is constructed, and the matrix being full rank indicates the structural identifiability of the model [34]. More detail on this analysis is included in previous work by the authors [21], and the algorithms are discussed here [35].

### Practical identifiability analysis

Practical identifiability analysis examines the uniqueness of parameters based on the experimental data. Profile likelihood-based methods consider one model parameter (e.g., $\psi_{i}$) as fixed at a given value and then find the maximum likelihood estimation of the rest of the parameters ($\psi_{j},\;\forall j\neq i$) in Eq 14, i.e.,

$$\mathrm{PL}\left(\psi_{i}\right)=\max_{\psi_{j\neq i}}\ell (\Psi ).$$ (16)

Note that Ψ is a vector of all model parameters defined by Ψ=[Θ,ξ]. This process is systematically repeated for different values of the fixed model parameter $\psi_{i}$. Then, the confidence interval for each parameter is defined as the set,

$${\mathrm{CI}}_{PL}\left(\psi_{i}\right)=\left\{\psi_{i}|PL\left(\psi_{i}\right)\geq \ell_{\max}-\Delta_{\alpha }\right\},$$ (17)

where $\ell_{\max}=\max_{\Psi }\ell (\Psi )$ is the maximum log-likelihood and $\Delta_{\alpha }$ is the *α*-quantile of the $\chi^{2}$ distribution with one degree of freedom [36,37]. Parameters with finite confidence intervals are deemed as practically identifiable.

## Results

### Structural identifiability analysis

We first performed structural identifiability analysis on all candidate models (StructuralIdentifiability.jl [35,38,39]). This analysis assesses the uniqueness of the inferred model given an infinite number of noiseless observations. The observables for performing this analysis are the same as the ones in the objective function. Results showed that all models are globally structurally identifiable.

### Model-based design of experimental protocols

In the context of ODE-based modeling of dynamical systems, an important aspect is the characteristic time scale of the ODE, i.e., the time required for the observables to vary significantly [40]. In simpler terms, we need to determine the sampling period required to infer an accurate model. We use previously published experimental observations on the iPSC differentiation process to guide the sampling period selection; Jacob et al. suggest testing DE efficiency every 24 hours [41]. Here, we perform a grid search around the 24-hour period (e.g., 12-, 24-, 48-, 72-, 96-, and 120-hour periods), to find a sampling period that minimizes the distance between assumed parameters, $\hat{\Theta }$, and inferred parameters, $\Theta^{*}$, using model-based design of experimental protocols [21].

The process starts by choosing a model with initial conditions consistent with our experimental setup. The assumption is that Eq 2 with Gompertz growth and parameter values in S1 Table ($\hat{\Theta }$) provide an appropriate description of iPSC differentiation, which creates qualitatively similar dynamics to prior experiments. Using the model and associated parameters, we generate synthetic data at a specific sampling period (e.g., 12-hour period) for all initial conditions for the observables, i.e., cell populations. Gaussian noise is then added to the data sampled as,

$$N_{j}(t_{k})=n_{j}(t_{k})(1+\epsilon \eta_{i}(t_{k}))\;\eta_{j}(t_{k})∼𝒩\left(\mu =0,\sigma^{2}=1\right)$$ (18)

where, 𝜖 is the noise level, $n_{j}(t_{k})$ represents the simulated value of observable *j* at time *t*_*k*_, and 𝒩 is the Gaussian probability distribution function.

Solving the minimization problem in Eq 14 results in the inferred structural parameters ($\Theta^{*}$). This process is then repeated with other sampling periods to perform a grid search (e.g., 24-, 48-, 72-, 96-, and 120-hour periods). We define a relative distance (error) measure between the inferred and assumed parameters as,

$$e=\frac{\left|\hat{\Theta }-\Theta^{*}\right|}{\left|\hat{\Theta }\right|}.$$ (19)

**Fig 2:**
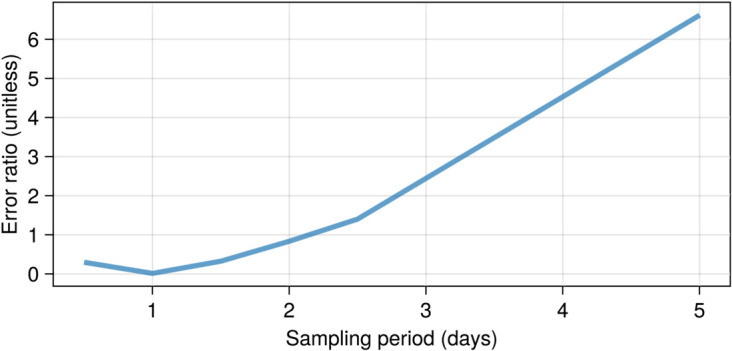
Parameter inference errors for different sampling periods. The best sampling period for our five-day experiment is daily.

Fig 2 shows the resulting error in parameter inference as a function of the sampling frequency for the observables using ε=0.3. For estimating parameters for the set of model proposals considered here, taking individual live and total dead cell population measurements every 24 hours results in a 1.50% error. Since neither more nor less frequent measurements improve the parameter estimates, we selected 24 hours for a five-day experiment as the sampling period for our iPSC differentiation experiments.

### Running and postprocessing the experiments

Two experiments were run with 900 000 and 300 000 cells per well initial plating populations (N = 4 and N = 2, respectively). Individual live cell populations from these experiments and total dead cell populations are collected. Experiments were conducted as described in the Experimental setup section.

### Model inference using *in vitro* data

We inferred the parameters for the 27 candidate models. The candidate models are the permutations of the three lineage models, the three growth models, and the three error models. Note that M1, M2, and M3 have 5, 8, and 12 parameters, and the exponential growth model does not add extra parameters, while the other growth models add one parameter of $n_{\max}$. As seen in Eq 11, the error models are additive, proportional, and combined, adding one, one, and two error parameters, respectively.

Model inference was done using the calibration dataset, which includes 48 data points from six replicates. For every replicate, we randomly assigned three time points to calibration, one to model selection, and one to the final test set, ensuring that all states from the same time point were kept together. This stratified split (i) ensures an even distribution of replicate-level systematic noise across all data subsets, (ii) prevents data leaks between different observables at the same time point, and (iii) reflects common laboratory situations where experimental data from a given day may be missing. The 60%-20%-20% split ratio (a) supplies enough observations to fit the practical identifiable model parameters, (b) leaves a dedicated selection subset large enough (16 data points) to rank 27 model candidates, and (c) preserves an equally sized test subset for an unbiased evaluation.

Optimization runs were conducted over a search space spanning multiple orders of magnitude, defined as a hypercube with a dimension equal to the number of parameters, with each parameter range [$10^{-4},10^{2}$] for rates (unit being $d^{-1}$), [0,1] for ratios (dimensionless), and $\left[n_{\max}^{u}/25,n_{\max}^{u}\right]$ for $n_{\max}$. The upper bound maximum cell population, $n_{\max}^{u}$, is the maximum number of cells if a monolayer covers the entire area of the well plate floor and is estimated assuming circular-shaped cells with a diameter of 15 μm. A total of 100 starting points for the optimization were selected using a maximin Latin hypercube [42] to ensure comprehensive coverage, amounting to 100×27=2700 optimization runs. Each optimization run used a variant of adaptive differential evolution, technically named “DE/rand/1/bin with radius-limited sampling” [43,44] (BlackBoxOptim.jl package [45]). Then, the inferred parameters are further optimized using the Nelder-Mead algorithm [46] (NLopt.jl [47]).

We created a modified loss, loss over common states (LOCS), to provide a uniform foundation for comparing different models, each with unique observables. As the name suggests, this loss uses the common states in the M1, M2, and M3 lineage models. This is equivalent to the M1 loss, defined by Eq 14, for only the populations of live iPSCs, live DE, and total dead cells. LOCS is necessary since the loss functions used for model calibration have more states for more complex models, making comparing the models, i.e., model selection, impossible. Hereafter, we refer to LOCS simply as “loss” in this section.

M1 models are at an advantage when comparing loss values on the training dataset, as observed by S1 Fig. Loss includes the same states that M1 models are trained on but are a subset of states for M2 and M3 models. This means model selection based on training data results in the M1 model being compared based on its training data, while the M2 and M3 models are compared based on a subset of their training data.

This advantage has been mitigated by using a selection dataset since now only the states favor the M1 model, not the specific data points. The mitigation is to the point that four models, logistic/Gompertz M2/M3 with combined error, have lower negative log-likelihood values than the best M1 model, meaning those are better models based on this criterion. This analysis suggests that the further non-linearity introduced by the lineage models helped better capture the evolution of the three state variables (S2 Fig).

**Fig 3:**
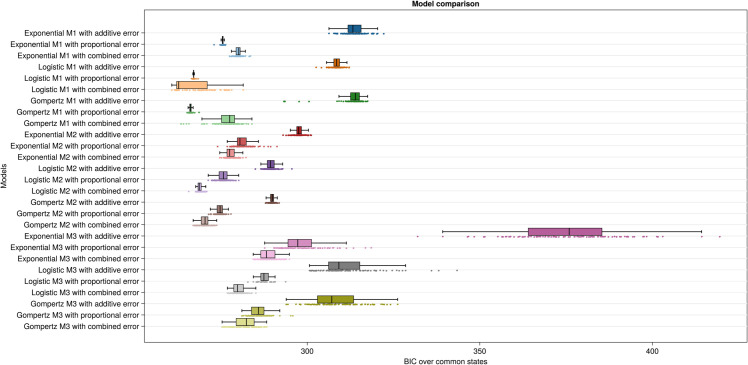
Model comparison based on BIC over common states. The rows show the 27 inferred models, with colors and shades representing structural and error models, respectively. Each row is derived from 100 parameter calibrations, each inferred from different initial guesses. The x-axis presents BIC over common states values as discrete points, where a lower value indicates better model performance. These values are collectively summarized in the form of a boxplot.

Then, loss using the selection dataset is used to calculate the Bayesian information criterion (BIC), defined as,

$$\text{BIC}=k\ln(m_{s})-2\ell (\Theta ,\xi ),$$ (20)

where *k* is the number of inferred parameters, and *m*_*s*_ is the number of observations in the selection dataset. When model complexity is penalized using BIC, the aforementioned models lose, and the best two models become logistic M1 with combined error and Gompertz M1 with combined error, respectively (Fig 3). This means the prediction capability of these models are not to the level that would justify the further model complexity. Another observation is that exponential growth always has the highest BIC value in all three lineage models, exemplifying its lower prediction capability. Comparing the error models, Fig 3 indicates that the best error model is the combined error, followed by the proportional error model.

To choose between logistic and Gompertz growth in the case of lineage model M1 with combined error, we look at S3 Fig and S4 Fig. S3 Fig depicts the histograms of the inferred parameters for M1 with logistic growth, corresponding to 100 different initializations of the optimization problem. Additionally, the orange dots indicate the best values of the inferred parameters, corresponding to the lowest loss values reached over the 100 parameter estimations. As depicted, the inferred values for most parameters are far from the modes of the respective histograms, pointing at multiple optima, with more than 40 of them having ${𝗉}_{sd}\approx 1$, implying no connection between the two populations of cells, i.e., no differentiation. S4 Fig implies that the case is different for the Gompertz growth model, and now all the histograms are unimodal, showing that most of the inferred parameters are in the same vicinity. Here, all but two inferred parameter values are near the modes. Furthermore, this figure shows lower dispersion for this model and more consistency in the inferred parameters. As mentioned before, ${𝗉}_{sd}$ shows the portion of the cells that go through renewal, so one of the insights from this model can be, contingent on the correctness of this model, the same ratio of the iPS cells go through differentiation as self-renewal (Table 1). Finally, looking at the confidence intervals for Table 1 and S2 Fig, it is evident that confidence intervals for the logistic growth model are larger, showing lower certainty in the inferred parameters (note that the confidence intervals are calculated in the next step). The evidence above supports the selection of Gompertz M1 with combined error as the best model.

**Table 1:** Inferred parameters of the Gompertz M1 model with combined error. Here, the empty unit rows stand for dimensionless.

Parameter	Value	Confidence interval	Unit
$\beta_{s}$	10.119	(5.1404,19.085)	${\text{day}}^{-1}$
${𝗉}_{sd}$	0.49823	(0.48376,0.51249)	
$\delta_{s}$	0.52186	(0.0033041,1.2024)	${\text{day}}^{-1}$
$\beta_{d}$	0.1881	(–0.095345,0.79051)	${\text{day}}^{-1}$
$\delta_{d}$	0.47299	(0.36077,0.6344)	${\text{day}}^{-1}$
$n_{\max}$	718.57	(394.64,1978.7)	${\text{cell mm}}^{-2}$
*a*	116.54	(88.265,155.68)	${\text{cell mm}}^{-2}$
*b*	0.34287	(0.2683,0.45133)	

**Fig 4:**
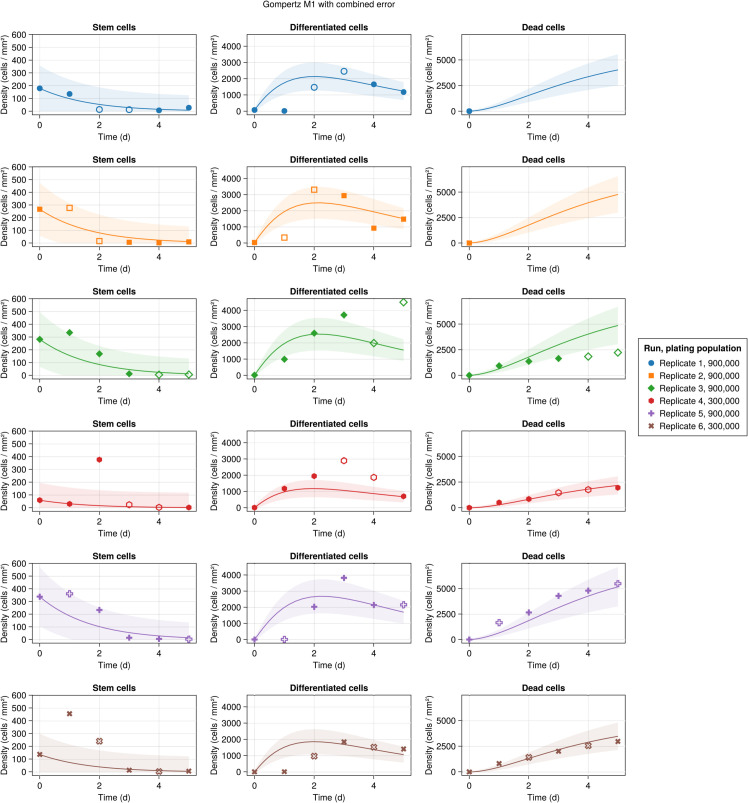
Experimental observations versus the model predictions for the M1 model with Gompertz growth. Solid lines represent model predictions, while shaded areas indicate the associated standard deviations derived from the error model. Also, solid and hollow markers indicate the training and selection/test datasets, respectively.

Model predictions versus experimental data are shown in Fig 4 with different markers/colors representing different replicates. Solid markers show the training dataset, while the hollow markers indicate the selection and test datasets. The curves represent the inferred structural model, while the bands show the standard deviation of the model. As can be seen, there is a good match between observations and the model predictions at most time points. It is also worth noting that for the iPSC, DE, and dead cell populations, respectively, only 13%, 43%, and 30% of the inferred points are outside the standard deviation of the model, which are comparable to the accepted value for a Gaussian distribution (approximately 32%). The highest deviation between the model and prediction appears on days 1 and 2 of the iPSC population. This is partly due to experimental observations on these two days that significantly deviate from the general trend of the data. Specifically, in some replicates, there is an initial increase in iPSC populations that cannot be captured by the structural model used in this work. This initial increase typically coincides with a stalling of growth in the DE population, which may suggest a lag in differentiation activation or a time-dependent differentiation ratio ${𝗉}_{sd}$ that starts at around 1 and decreases after 24 hours.

**Fig 5:**
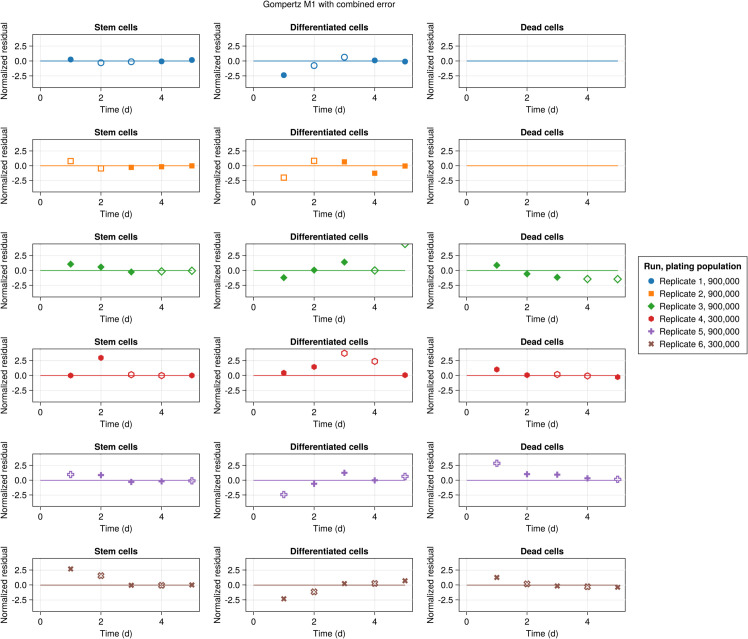
Normalized residuals over time for the M1 model with Gompertz growth. Solid and hollow markers denote the training and selection/test datasets, respectively.

Normalized residuals for each replicate are illustrated by Fig 5 where different markers/colors indicate replicates and solid and hollow markers represent training and selection/test datasets. The figure qualitatively indicates that the model residuals are not correlated. Further, the Durbin-Watson test [48] quantitatively assesses the null hypothesis of no correlation in the errors. This test yields a value of 2.01, which does not indicate residual correlation, although the small sample size is noted [32].

### Practical identifiability analysis

**Fig 6:**
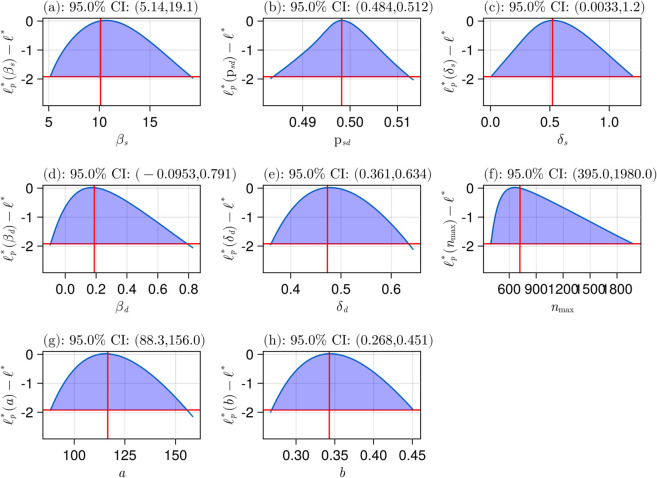
Likelihood profile for Gompertz M1 model with combined error. Each x-axis corresponds to a parameter in the model, and the y-axis is the log-likelihood value.

To assess whether the parameters of the selected model, Gompertz M1 with combined error, are uniquely determined by the data, we perform profile likelihood-based practical identifiability analysis (ProfileLikelihood.jl [49]). Note that this step differs from structural identifiability analysis, as it also incorporates experimental data. Fig 6 shows the resulting likelihood profiles for each of the parameters. Note that the red vertical and horizontal lines correspond to the inferred parameters, and $-\Delta_{\alpha }$ in Eq17 with 95% confidence, respectively. The intersections of the curve and the horizontal line show the lower and upper bounds, summarized in Table 1. The finite resulting confidence intervals for all estimated model parameters confirm the practical identifiability of the model based on our experimental data [37]. Note that the confidence interval for $\beta_{d}$ contains zero, suggesting that the effect may not be statistically significant based on the collected data, as the parameter potentially can be zero.

For comparison, S2 Fig and S5 Fig show the results of running the analysis on logistic M1 with a combined error model. As mentioned, the confidence intervals here are larger, indicating lower confidence in each of the inferred parameter values, which is another reason for selecting the Gompertz M1 model.

### Goodness of fit

The normalized root-mean-square error (NRMSE) of the inferred model was calculated using the validation dataset. It is defined as,

$$\text{NRMSE}=\frac{1}{\max(𝐍)-\min(𝐍)}\sqrt{\frac{1}{m}\sum_{j=1}^{n_{z}}\sum_{k=1}^{n_{k}}(N_{j_{k}}-n_{j}\left(\Theta ,t_{k}\right){)}^{2}}.$$ (21)

In this equation, **N**, *n*_*z*_, *n*_*k*_, and *m* are the experimental data, the number of data points in each state, the number of states, and the total number of data points, respectively [50]. The NRMSE of the inferred model on the validation dataset is calculated to be 26.4%. This NRMSE value is below a commonly used threshold of 30% [51], and thus we consider the model validated and sufficiently accurate for its application in support of iPSC differentiation to DE.

### Global sensitivity analysis

A global sensitivity analysis of the selected model, Gompertz M1 with combined error, was performed. This analysis highlights the significance of each parameter in determining model predictions. Specifically, we calculated the sensitivity of the iPSC, DE, and dead cell populations with respect to the structural model parameters. For this purpose, we use Sobol’s method [52] with 40 000 Monte Carlo samples from the bounds in Table 1. First-order Sobol indices rank the importance of each condition alone, while total-order Sobol indices also include parameter interactions (GlobalSensitivity.jl [53]).

**Fig 7:**
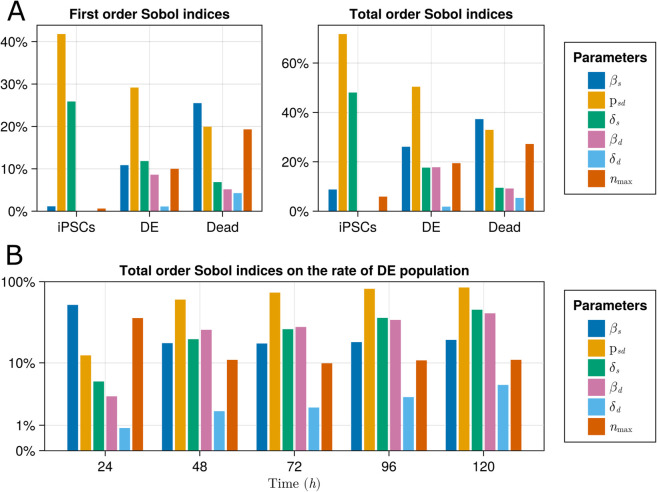
Global sensitivity analysis on Gompertz M1 model. (A) GSA on day 3 of the model states. (B) Time evolution of the total order Sobol indices of the rate of DE population to model parameters.

Fig 7A shows the resulting Sobol indices for the plating population of 900 000 cells per well at time *t* = 72 hr as predicted by our model. The first-order and total-order Sobol indices are observed to be qualitatively consistent, implying that the interactions are less pronounced than the first-order effects. Also, it can be observed that the proliferation and death of DE have no effects on the population of iPSCs, as seen directly from Eq 2. Furthermore, the iPSCs population is mainly driven by the differentiation ratio, ${𝗉}_{sd}$, and their death rate, $\delta_{s}$. This is expected since the iPSCs population is decline-dominated, which is the effect of $\delta_{s}$ and ${𝗉}_{sd}$ as stated in Eq 2; both are parameters in the terms with a negative sign in the equation for iPSCs population.

Further, it is observed that the population of DE is predominantly driven by differentiation ratio, ${𝗉}_{sd}$, and iPSCs proliferation rate, $\beta_{s}$. This is explained as observed by the experimental data and the inferred model (Fig 4); the population of DE drops after the iPSC population becomes small, suggesting the main driver of the DE population is not DE renewal and instead is the differentiation of the iPSCs.

Fig 7B shows the time evolution of total-order Sobol sensitivity indices throughout the experiment based on the inferred model. It can be observed that the sensitivity is fairly consistent for the DE population, and the role of $\beta_{s}$ decreases due to the depletion of its population. Also, as the DE population is becoming decline-dominated, the effects of the death rates become more dominant.

## Applications

The main driver of mathematical model development is in the utility of the inferred model for rapid prototyping of *in silico* experiments to explore different protocols and culture environments [54]. In this section, we conduct *in silico* experiments to determine the optimal culture time and plating population. Then, we will use the model to explore the effect of modifying the experimental protocol by adding ROCKi to the cell growth media. These experiments can be used *a priori* to the *in vitro* models to form quantifiable hypotheses and better design and direct cell culture protocols.

### Effect of plating population

When starting to experiment with iPSCs, one of the first questions is the optimal plating population and differentiation timespan for DE induction [24]. The validated model is used to perform a grid search on model initial conditions to explore the effect of different plating populations and to provide insights into local trends of varying this factor. This grid search allows the simultaneous optimization of the differentiation timespan.

Before running the *in silico* experiments, there is a need to perform a mapping between the controllable experimental parameter, in this case, the plating population, and the populations used as initial conditions for the models. The inferred model explains the dynamics through day 0 to day 3 of the growth protocol shown in Fig 1A using the measured iPSC and DE populations on day 0 but did not connect those values to the plating populations (day –1 populations, a day before DE induction Fig 1A). The first step is to create a population mapping between day –1 and day 0 in Fig 1A.

For this purpose, we fit two linear models with ordinary least squares (using GLM.jl [55]) to map the plating population, which is entirely iPSC, to day 0 populations, which are iPSC and DE populations.

$$n_{s_{0}}=\beta_{1}n_{p}+\beta_{0_{1}},\;\;n_{d_{0}}=\beta_{2}n_{p}+\beta_{0_{2}}.$$ (22)

Note that *n*_*p*_ is the plating population of iPSCs on day –1, and $n_{s_{0}}$ and $n_{d_{0}}$ are the day 0 populations of iPSCs and DE, respectively. The top plot in S6 Fig depicts the initial conditions used in our models (same as used in Fig 4), and the line shows the one-dimensional space we search to observe the effect of the plating population. The lower plots depict the two fitted mappings, which link the experimentally controllable plating density to the initial conditions required by the mathematical model. We adopted an affine form because it is a parsimonious model that captures a single piece of prior knowledge that only a fraction of plated cells attach and survive the first 24 hours after plating, called plating efficiency [56], while a constant intercept accounts for systematic loss or measurement bias [57]. More sophisticated mappings can therefore be revisited once richer data becomes available.

Derived outputs are used to quantify the effect of different plating populations. The maximum density of DE is named $n_{d_{\max}}$, and its corresponding time is called $T_{\max}$. Two other variables are yield per input cell, defined as the ratio of DE population over plating population, and DE ratio, which is the ratio of cells that are differentiated to the total cell population. The mathematical definitions for these variables are as follows,

$$T_{\max}=\arg\max_{t}n_{d},\;\text{Yield per input cell}=\frac{n_{d}}{n_{p}},\;n_{d_{\max}}=\max n_{d},\;\text{DE ratio}=\frac{n_{d}}{n_{s}+n_{d}}.$$ (23)

**Fig 8:**
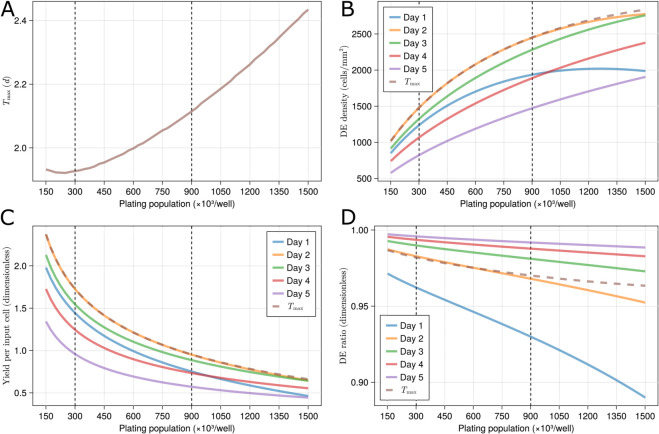
The effect of different cell plating populations. Each figure illustrates the effect of the plating population on one of the derived outputs as predicted by our calibrated model. (A) Best DE differentiation timespan, $T_{\max}$. (B) DE density, *n*_*d*_. (C) Yield per input cell, $n_{d}/n_{p}$. (D) DE ratio, $n_{d}/\left(n_{s}+n_{d}\right)$. Standard deviations for plots (B) and (C) are depicted in S7 Fig and S8 Fig, respectively.

Given the multiple output variables (i.e., objectives) that may be used to evaluate cell culture protocols (Eq 23), it follows that there is no single optimal DE differentiation timespan and plating population; instead, it depends on which objective is used for assessment. Here, we discuss three objectives for maximizing the derived outputs of DE density, yield per input cell, and DE ratio. Fig 8 illustrates the derived outputs when investigating the plating populations at different time points as predicted by the model. The vertical dashed lines across all the figures correspond to plating populations of 300 000 and 900 000 cells per well, which are the two plating populations used for model inference.

#### DE differentiation timespan.

According to Fig 8A, to maximize DE density, the optimal timespan for DE differentiation lies between 1.92 and 2.43 days, with longer durations required for higher plating populations. This does not indicate slower cell growth at higher plating populations. As observed in Fig 8B, increasing the plating population results in higher DE densities at a fixed time point. Also note that culturing cells for three days does not have a substantial negative impact, as seen by Fig 8B; the average drop in DE population between day 2 and day 3 is 6.8%±2.4% (S7 Fig, the standard deviations are calculated using the error model, as was done for Fig 4). The average drop in population between days 3 and 4 is more substantial, at 16.0%±5.5%. This analysis suggests that the differentiation protocol needs to be run for two days, and extending it to three days does not have a substantial negative effect on producing the highest population of DE at the end of the DE induction step.

As illustrated by Fig 8C, the optimized differentiation timespan would be the same if the optimization objective is maximizing the yield per input cell. Fig 8D predicts that using the protocol, 95% of the cells are DE after two days, implying a substantial proportion of the cells differentiated to DE. It also suggests that when maximizing the DE ratio is the aim, DE ratios at day 3 are about 1% better than the ratios at $T_{\max}$ (from 97.5% to 98.5%), meaning that if a higher DE purity is desirable, it is best to continue the differentiation protocol up to 3 days instead of that suggested by $T_{\max}$ alone. In summary, when optimizing the differentiation timespan by maximizing the DE population or yield per input cell, a duration of around two days (corresponding to the plating population of 600 000) is predicted to be the ideal period, while three days of differentiation is implied to be ideal when optimizing for DE ratio.

#### Plating population.

Fig 8B also explores the effect of the plating population on daily DE populations, suggesting $n_{d_{\max}}$ increases with plating populations. Fig 8B further suggests the downward concavity of DE density over the plating population, indicating that the yield per input cell is decreased at higher plating populations, as also depicted by Fig 8C. This figure implies that the system’s efficiency decreases as the plating population increases, with yield per input cell dropping from 1.72±0.74 to 0.95±0.38 as plating population is increased from 300 000 to 900 000 cells per well (S7 Fig). This might be caused by population growth being constrained by a lack of space, nutrients, growth factors, or excess waste products.

These analyses suggest that plating populations of 300 000 to 900 000 cells per well are acceptable populations for the differentiation protocol; with lower certainty, this bound is extended to lower/higher values. The plating population of 900 000 cells per well optimizes for DE density in terms of the number of wells, i.e., when the growth media is expensive. On the other hand, the model predicts that 300 000 cells per well optimizes for yield per input cell (Fig 8C) and DE ratio (Fig 8D). Yield per input cell focuses on the higher costs associated with the number of iPS cells. This analysis illustrates how different objectives can be optimized by incorporating *in silico* modeling into cell differentiation studies.

Four additional replicates (not used for model calibration, selection, or the previous validation) were performed to validate this *in silico* experiment. Two replicates had a plating population of 300 000, and two had a population of 450 000. For these replicates, we calculated the maximum yield per input cell value for each replicate. The markers in S8 Fig denote the experimental data, and as shown, only one out of four data points lies outside the model standard deviation, which is similar to a Gaussian distribution (approximately 32%). Note that the model is conservative and tends to underestimate maximum yield per input cell. Additionally, the model further underestimates the values for the 450 000 plating population, which may be due to the trend not being as monotonic as the model predicts. These results suggest that a larger dataset with more initial plating populations would help the model better capture the influence of this parameter.

### Effect of ROCK inhibitor

Y-27632 is a Rho-associated protein kinase (ROCK) inhibitor for ROCK1 and ROCK2 in the Rho/ROCK pathway that competes with adenosine triphosphate (ATP) for binding to the catalytic site [58–60]. Kamei et al. [61] studied the effect of adding Y-27632 to the DE induction medium, suggesting it would increase the survival of iPS cells by decreasing the death rate while not affecting proliferation. Some iPSC differentiation to DE protocols include Y-27632 in day one of DE induction growth medium [62] while some for the entire differentiation to DE process [61]. Here, we utilize the developed model to run some hypothetical scenarios to study the effect of Y-27632 on the first 24 hours of our protocol.

Since the growth media composition is protected as a trade secret, we assume that Y-27632 is not in the media and check what might happen if added for the first 24 hours. First, we perform GSA on the model parameters $\delta_{s}$ and $\delta_{d}$ in the range of $[0,1]\cdot \delta_{j}^{*}$, with $\delta_{j}^{*}$ indicating the inferred values, to see the relative importance of the two death rates on the day 1 DE population. In other words, we vary the death rates between zero and the inferred values to capture their importance. This analysis states that the iPSC death rate can explain 85% of the population variance, suggesting that the day 1 DE population is dominated by the stem cell death rate, although the DE death rate contribution is not negligible. So, the objective becomes studying the effect of different reduced values of $\delta_{s}$ and $\delta_{d}$ on $n_{d_{\max}}$ and $T_{\max}$ defined in Eq 23.

**Fig 9:**
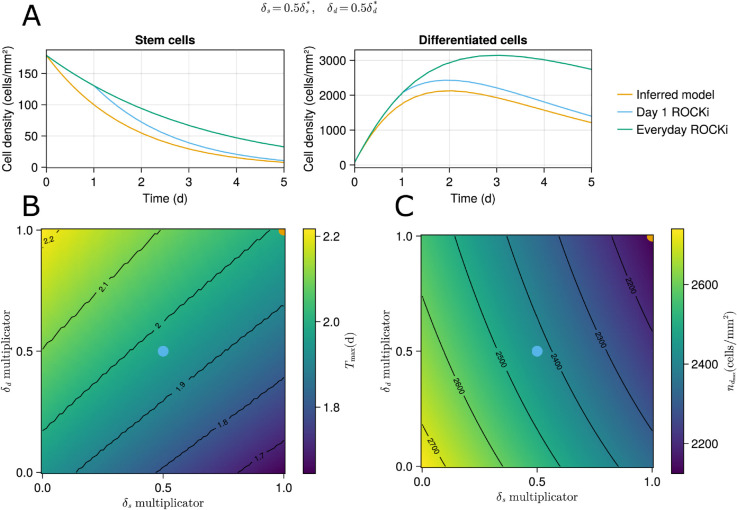
Effect of ROCKi. (A) Two different modes of exposure to ROCKi, the one-day exposure is better supported by the literature. The figures illustrate the effect of ROCKi, assuming the death rates are halved for 1 day (blue curves) and throughout the entire time (green curves). (B, C) The effect of one-day exposure to ROCKi, assuming different multiplicators for the death rates, on the maximum DE density and its corresponding time. (B) Best DE differentiation timespan, $T_{\max}$. (C) Max DE density or $n_{d_{\max}}$.

Fig 9A depicts the time evolution of the effect of ROCKi on the population dynamics with the inferred model without ROCKi exposure, day 1 ROCKi exposure, and 5-day ROCKi exposure shown with orange, blue, and green curves, respectively. For this figure, it is assumed that the ROCKi would halve the death rates, $\delta_{s}$ and $\delta_{d}$. It is observed that one-day and culture-long exposures of ROCKi would increase the maximum DE population by 14% and 47%, respectively. Note that all these models correspond to the median initial condition corresponding to the plating population of 900 000 cells per well. Short-term exposure to ROCKi increases initial adhesion and survival of dissociated hESCs while prolonged exposure decreases prolonged attachment and viability of hESCs [63]. Since the adverse effect on growth is not accounted for in the model, we will continue the analysis with only the 24-hour exposure model.

To our knowledge, the literature lacks quantitative studies measuring the effect of Y-27632 on the death rates of iPSCs and DE. So, a grid search over the interval $[0,1]\cdot \delta_{j}^{*}$ is performed, and derived outputs are computed. Fig 9B shows $T_{\max}$ over different values of the death rates for the first 24 hours as predicted by the model. The cyan and orange dots on the plot correspond to the cyan and orange curves in Fig 9A. So, the orange dot indicates no ROCKi effect, and the origin indicates when both death rates are zero for the first 24 hours. It is observed that decreasing both death rates by the same ratio would have a limited effect on $T_{\max}$, thus suggesting that ROCKi would not alter the optimal differentiation timespan. S9a Fig and S9b Fig correspond to the lowest and highest initial conditions used for model inference, further corroborating the low correlation between $T_{\max}$ and the death rates. Fig 9B also suggests that the greatest change in $T_{\max}$ is when one of the multiplicators is zero, and the other is one, corresponding to one of the death rates not being affected and the other becoming zero. In this case, the change in $T_{\max}$ is about 8 hours. Finally, note that it is already discussed based on the model (Fig 9A) that around the optimal time, the DE population is stable, e.g., the blue curve does not change radically between time points 1.6 and 2.4 days.

Fig 9C depicts the effect of adding ROCKi to the medium for the first 24 hours on maximum DE density $n_{d_{\max}}$ as predicted by the model. It is suggested that Y-27632 can increase the maximum population up to 28%. Further, S9c Fig and S9d Fig imply that at lower and higher day 0 populations, this effect ranges between 39% and 16%, respectively, suggesting that Y-27632 is more effective at lower plating populations.

## Discussion and conclusion

This paper presents a mathematical model of iPSC differentiation into DE population dynamics. We initially designed the experiment length and sampling period using model-based design of experimental protocols [21]. We then ran experiments with measurements of individual live populations and total dead cell populations, which were used to search over 27 models resulting from the permutations of three lineage, growth, and error models. Model selection was performed over the model space, and the practical identifiability of the selected model was confirmed, supporting the uniqueness of the inferred model parameters. Global sensitivity analysis ranked the importance of model parameters, implying the dominance of the differentiation process on the dynamics of the DE population. Finally, the model was validated using NRMSE on the validation dataset, achieving a value of 26.4%, below the threshold of 30% [51]. This model will be a valuable tool for furthering our understanding of the differentiation process of iPS cells to DE. As such, we will be able to optimize the process for various applications depending on the target cell type and stage of differentiation after DE. As mentioned, directed differentiation of iPSCs towards DE is essential to regenerative medicine, as all endoderm derivatives, such as the lungs, thyroid, liver, and pancreas, undergo this initial step. Modeling this step facilitates the development of disease treatments and provides critical insights into developmental biology.

We leveraged the validated model to analyze and optimize the differentiation protocol, focusing on a) plating population, b) differentiation timespan, and c) addition of ROCKi to the culture medium for the initial 24 hours. These *in silico* experiments suggested that increasing the plating population increases the final DE population, although it decreases the system’s efficiency, i.e., the yield per input cell, meaning that the plating population of 300 000 cells per well would be more desirable. The yield per input cell can be a crucial variable for optimization when dealing with rare and expensive stem cells. Handling multiple objectives simultaneously is demonstrated by Olofsson et al., where they utilized Bayesian multi-objective optimization to maximize the neotissue growth while minimizing the operating costs [64].

Using our model, we suggested that the optimal time for differentiating iPSCs into DE based on the yielded population is between two and three days. A question that still needs to be addressed, and the current model is insufficient for, is whether culturing for less than three days, which is the time recommended for the STEMdiff kit, has any adverse effects on later stages of the differentiation process. This is important as the optimum yield of DE depends on the target cell in the differentiation protocol. For example, Hawkins et al. demonstrated that optimizing for the efficiency of DE at the end of the DE induction step yields the optimal differentiation timespan of 84 hours. On the other hand, when optimizing the efficiency of NKX2-1+ lung progenitor cells at day 15 of their protocol, the optimal DE differentiation timespan was lower and closer to 72 hours [24].

Another *in silico* experiment investigated the effect of 24 hours of Y-27632 ROCKi in the growth media on the population dynamics. Assuming the differentiation medium, STEMdiff Definitive Endoderm Kit, does not contain ROCKi, namely Y-27632, and based on experimental observations in the literature, we designed experiments to study the effect of ROCKi on differentiation to DE. The assumption regarding the effect of ROCKi on model parameters is that the death rates decrease during exposure and fully recover afterward. This maintains minimal assumptions while providing bounds for the ROCKi effect, eliminating the need to recalibrate the model using an additional set of experiments. The analysis suggested that ROCKi would not significantly affect the optimal culture time and could increase the maximum DE population by 39% and 16% for 300 000 and 900 000 cells per well plating populations, respectively. Further *in vitro* experiments are needed to validate these assumptions and observations, and to help quantify the effect of Y-27632 on death rates.

Different iPSC have variable responses when exposed to the same small molecules [65,66]. When applying an existing protocol to a new cell line, some aspects should be optimized, such as the plating population and differentiation timespan. The optimization can be augmented with *in silico* modeling as shown for the BU3 NGST iPSC line used in this work. The process also includes finding a new set of parameters and comparing them with Table 1, which helps to understand how the cell lines differ, i.e., the difference between the proliferation and death rates or differentiation ratios.

Another valuable feature of the *in silico* approach is its ability to provide a better understanding of the underlying biology of the differentiation process. As seen in the model development section, we initially assumed three different lineage models, two with transition states, before fully differentiating to DE. The transition states were assumed to express either one of the biomarkers of fully differentiated DE. The models developed in this work, based on our experimental data, do not support lineage models with transition states expressing CD184 or CD117. This finding is consistent with previous work by Green et al. [9], Chu et al. [67], and Li et al. [68], who also found that a transition state, mesendoderm, exists; however, the transitional cells express different biomarkers. Based on these observations, the inferred model can be improved by including a transition state for mesendoderm, which expresses Brachyury T (TBXT), MIXL1, EOMES, and GSC [67]. Then, a collection of mathematical models similar to the M2 lineage model can be calibrated, and a similar model selection protocol can be utilized.

As mentioned, the models are constructed on the assumption of finite, discrete intermediate stages in iPSC differentiation to DE. An alternative method is to define the differentiation process as cells moving in a multidimensional continuous space instead of between a few discrete stages. In this method, continuous transport equations would predict the movement of cells over a maturation coordinate such as age, size, and transcriptomic pseudotime [69]. The method used here yields parsimonious models, reducing the number of data points required to infer a practically identifiable model with FACS data sufficient for parameter estimation. In the future, using higher-frequency data collection supplemented by single-cell data to recover pseudotime, continuous transport equations can be inferred for a more detailed description of the differentiation process [69].

One limitation of the model is the underestimation of the dead cell count. This is due to the limitation in experimental data collection, where only the dead cells that are not lysed are counted. One solution is to modify data collection to perform real-time monitoring of cell culture, which would account for lysed cells [70]. Another method is to modify the maximum likelihood estimator (Eq 14) to follow a truncated Gaussian distribution for the dead cell population [71]. This would define a lower bound for the dead cells, but the rest of the populations can still follow a Gaussian distribution.

We acknowledge that the selection subset comprised only 16 data points. While this was sufficient to discriminate among the 27 candidates using BIC, a larger selection pool would lower variance in model ranking and further guard against overfitting. Future studies would include additional time points and plating densities to expand the selection set and to confirm the robustness of our model selection procedure.

In this work, we treat the ODEs as a model of the systematic within-experiment dynamics. Replicates are allowed to differ only through their initial conditions and the realized measurement noise, while all structural and error parameters are shared across experiments. Our stratified split assigns different time points from each replicate to the calibration, selection, and test sets; consequently, the test evaluation measures interpolation within each replicate’s trajectory rather than prediction for entirely new experiments. The scope of the generalization claims we make is therefore confined to within-experiment interpolation; assessing out-of-experiment predictive performance would require an explicit treatment of between-experiment variability [18].

Parameter pooling across replicates is appropriate when a common mechanism is assumed and improves the efficiency of estimating shared parameters. To mitigate potential pooling bias arising from between-experiment heterogeneity, we (i) use replicate-specific initial conditions taken from day 0 measurements, and (ii) evaluate goodness of fit on held-out time points drawn from each replicate, ensuring performance is assessed within each experiment. We acknowledge that if experiments differ systematically in ways not captured by initial conditions (e.g., batch effects), pooled estimates could in principle be biased. A hierarchical extension with random effects on selected parameters would more generally model between-experiment variability; however, such an extension increases complexity and data requirements and is therefore beyond the present scope [26,72,73].

The data used in the model development procedure are from experiments with 300 000 and 900 000 cells per well plating populations, which is the standard interval used in the literature [41]. In the future, the existence of a minimum population for the iPSCs to successfully differentiate into DE can be explored. This behavior cannot be captured with the growth models in our candidate models and requires growth models with the Allee effect [74]. Also, experiments with lower plating populations must be added to the dataset presented here for model selection. This enables the model to cover a more comprehensive experimental range and allows the search for a global optimum regarding plating populations.

Significant research has been conducted on establishing protocols for producing iPSC-derived DE populations [5,6,9,75]. The protocols typically include Activin A and low-level WNT signaling to support the activation of the NODAL pathway [1,76]. Commercial DE induction kits such as STEMdiff Definitive Endoderm Kit (StemCell Technologies), Gibco PSC Definitive Endoderm Induction Kit (Thermo Fisher Scientific), and StemXVivo Endoderm Kit (R&D Systems) are already available [77]. Due to wide adoption by the regenerative medicine community and the utilization of the STEMdiff media in many protocols for later-stage cells, e.g., alveolar cells [41], airway cells [78], intestinal organoids [79], thyroid epithelium [24], liver cells [80], this paper focused on DE induction using this media.

In the future, lab-made differentiation media with known nutrients and growth factor concentrations would help construct a mechanistic model that incorporates cell signaling pathways [81,82]. These models would have more states for different biomarkers, inhibitors, catalysts, and proteins, offering a higher degree of model explainability. Consideration of other components of the biochemical environment could also cause some model parameters, such as the maximum cell density, $n_{\max}$, to be independent of the growth medium and be based solely on contact inhibition [83]. Such model inference would also necessitate more measurements from the different states, such as activin A, to ensure a reliable and identifiable model. This can be achieved by conducting *in vitro* experiments with varying levels of nutrients, growth factors, and media replacement periods [21,84].

The differentiation protocol used in this work involves stepwise exposure to small molecules and growth factors to drive transcription factor activation. This would require tightly controlling the timing of exposures and seeding densities among other factors. An improvement is the utilization of synthetic genetic control, wherein a stable transcription factor is maintained by suppressing undesired lineages or adding feedback controls. For example, Ilia et al. [85] developed a system that uses a synthetic genetic circuit to generate a range of OCT4 expression levels during reprogramming of fibroblasts into iPSCs. They monitored OCT4 expression over time and found that the rare cells that successfully reach pluripotency express OCT4 at supraphysiologic levels. Similar systems may be useful for studying the dynamics of key transcription factors during the differentiation of iPSCs into DE. Similarly, Del Vecchio et al. [86] outlined a blueprint for a synthetic gene circuit with feedback control that could achieve stable transcription factor expression levels. It should be noted that their model does not account for dilution due to cell division, and therefore, careful optimization would be required to apply this blueprint to the directed differentiation of highly proliferative iPSCs. Similarly, synthetic genetic control may prove useful in limiting gene expression noise in directed differentiation protocols, ultimately yielding more efficient production of desired cell types [87].

## Supporting information

S1 Fig Model comparison based on LOCS on training data. (EPS)

S2 Fig Model comparison based on LOCS. (EPS)

S3 Fig Inferred parameters histograms for M1 model with logistic growth. The histograms show the distribution of the inferred parameter values from 100 optimization runs, and the orange dots show the inferred parameter values corresponding to the best (lowest loss) out of the 100 runs.
(EPS)

S4 Fig Inferred parameters histograms for M1 model with Gompertz growth. The histograms show the distribution of the inferred parameter values from 100 optimization runs, and the orange dots show the inferred parameter values corresponding to the best (lowest loss) out of the 100 runs.
(EPS)

S5 Fig Likelihood profile for M1 logistic model with combined error. Each x-axis corresponds to a parameter in the model, and the y-axis is the log-likelihood value.
(TIF)

S6 Fig Population mapping for day –1 plating population to day 0 iPSC and DE populations. Orange lines show the inferred mappings, and the blue dots show the experimental data used for the mapping.
(EPS)

S7 Fig The effect of different cell plating populations on DE density. Shades show the DE density standard deviation at days 2, 3, and 4.
(EPS)

S8 FigThe effect of different cell plating populations on yield per input cell.Shades show yield per input cell standard deviation at $T_{\max}$. The markers show the experimental maximum yield per input cell for four additional replicates.(EPS)

S9 FigThe effect of ROCKi on high/low bounds of the plating populations.(a) Best DE differentiation timespan, *T*_*max*_, for low plating population. (b) *T*_*max*_ for high plating population. (c) Max DE density, $n_{d_{\max}}$, for low plating population. (d) $n_{d_{\max}}$ for high plating population.(TIF)

S1 Table Model parameters for model-based experimental design of iPSC differentiation. (PDF)

S2 Table Inferred parameters of the Logistic M1 model with combined error. (PDF)
