# Supplementary figures and images for: In silico modeling of directed differentiation of induced pluripotent stem cells to definitive endoderm
Source: PLoS Comput Biol. 2025 Aug 21;21(8):e1013407. doi: 10.1371/journal.pcbi.1013407 (PMC12404646; doi:10.1371/journal.pcbi.1013407)

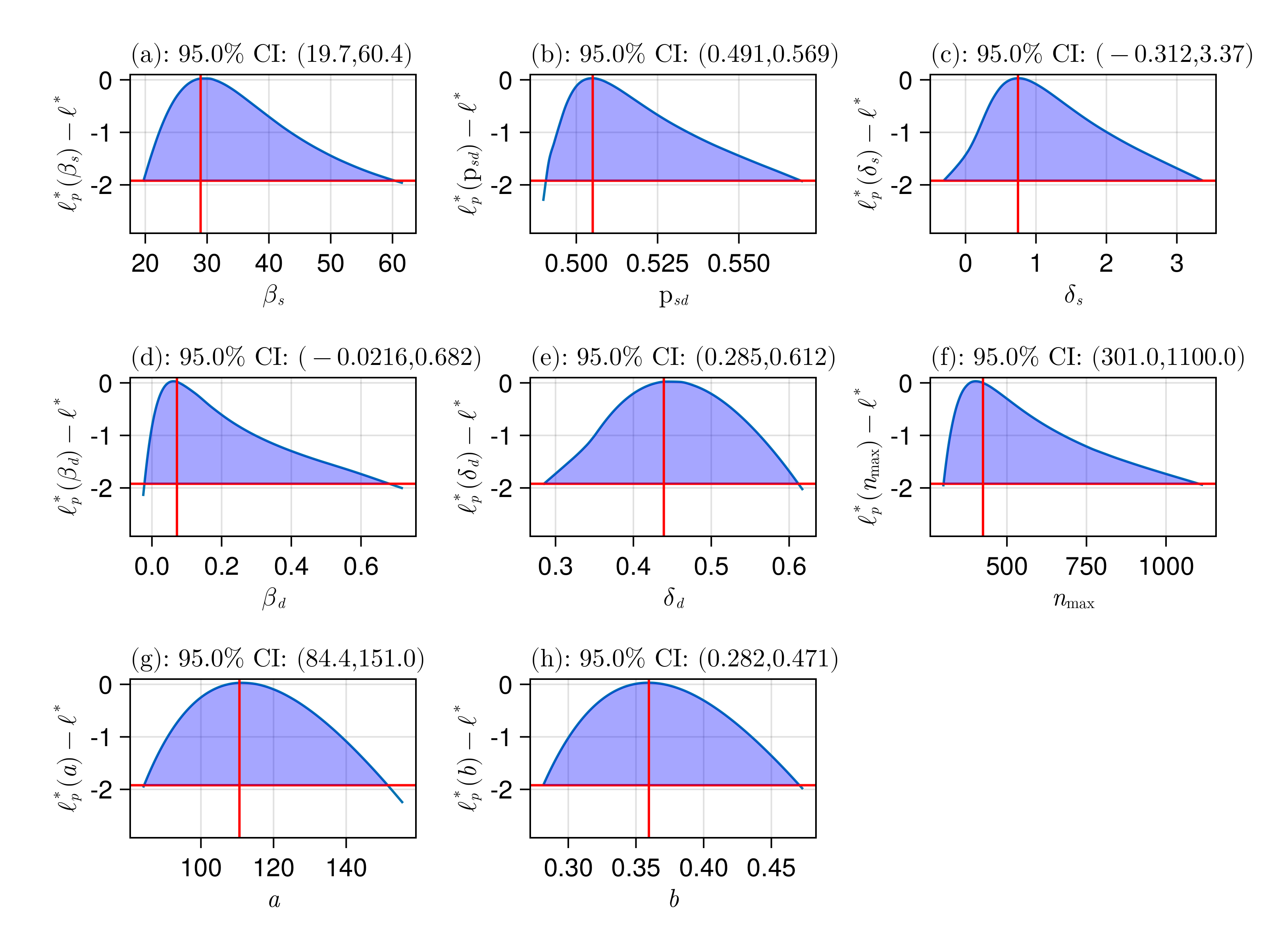

Supplement: S5 Fig — Each x-axis corresponds to a parameter in the model, and the y-axis is the log-likelihood value. (TIF) [file pcbi.1013407.s005.tif]

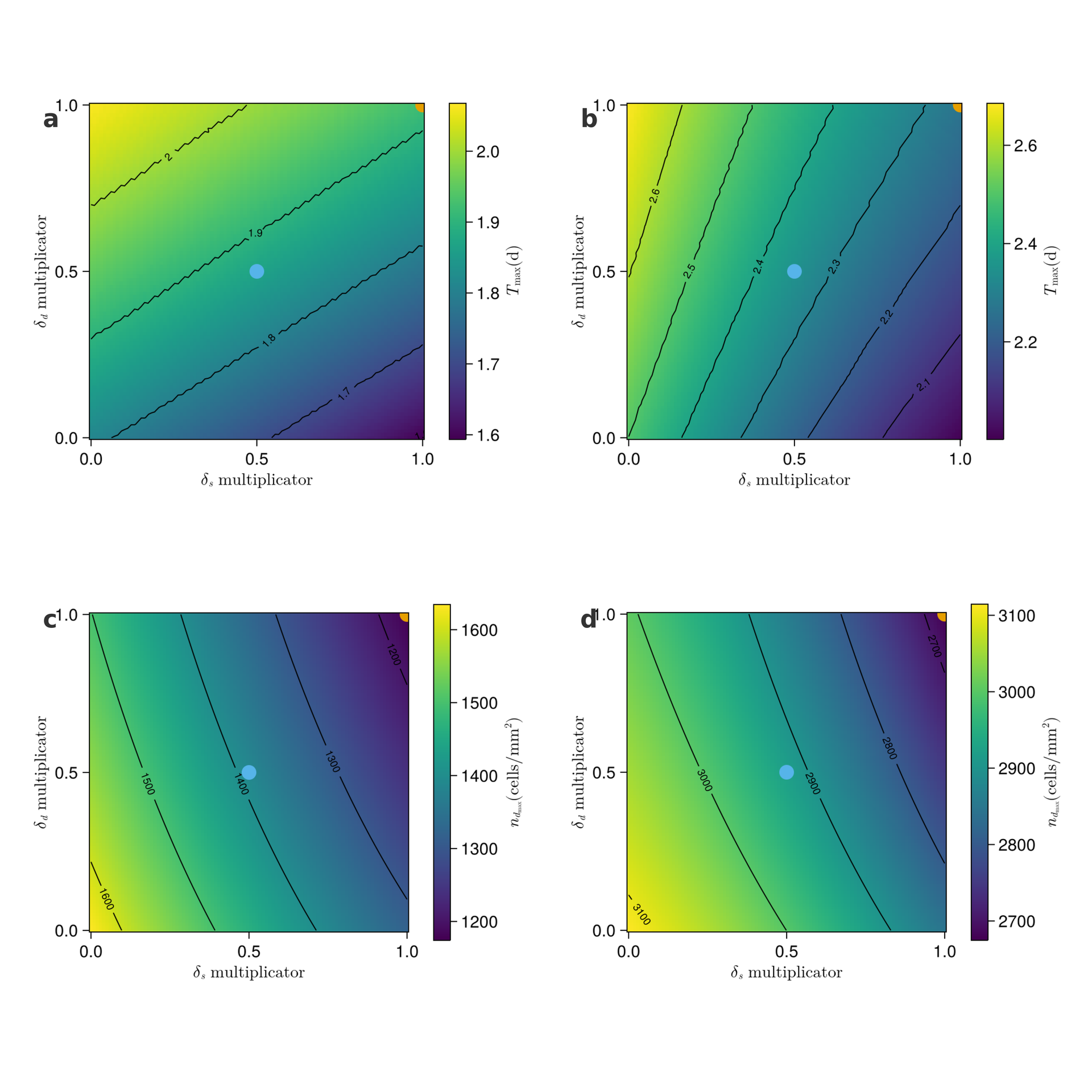

Supplement: S9 Fig — (a) Best DE differentiation timespan, Tmax, for low plating population. (b) Tmax for high plating population. (c) Max DE density, ndmax, for low plating population. (d) ndmax for high plating population. (TIF) [file pcbi.1013407.s009.tif]
