# Supplementary material for: In silico modeling of directed differentiation of induced pluripotent stem cells to definitive endoderm
Source: PLoS Comput Biol. 2025 Aug 21;21(8):e1013407. doi: 10.1371/journal.pcbi.1013407 (PMC12404646; doi:10.1371/journal.pcbi.1013407)
Supplement: S1 Table — (PDF) [file pcbi.1013407.s010.pdf]

Table S1: Model parameters for model-based experimental design of iPSCs differentiation.

Parameter	Value (p_0)	Unit
ε	0.3	dimensionless
β_s	1.0	day^{-1}
p_{sd}	0.5	dimensionless
δ_s	1.0	day^{-1}
β_d	1.0	day^{-1}
δ_d	1.0	day^{-1}
$n_{\max}$	500	cell mm^{-2}
