# Supplementary material for: In silico modeling of directed differentiation of induced pluripotent stem cells to definitive endoderm
Source: PLoS Comput Biol. 2025 Aug 21;21(8):e1013407. doi: 10.1371/journal.pcbi.1013407 (PMC12404646; doi:10.1371/journal.pcbi.1013407)
Supplement: S2 Table — (PDF) [file pcbi.1013407.s011.pdf]

Table S2: Inferred parameters of the Logistic M1 model with combined error.

Parameter	Value	(Lower bound, Higher bound)	Unit
β_s	28.951	$(19.705, 60.407)$	day^{-1}
p_{sd}	0.50505	$(0.49065, 0.56909)$	dimensionless
δ_s	0.74465	$(-0.31189, 3.3703)$	day^{-1}
β_d	0.071773	$(-0.021582, 0.68154)$	day^{-1}
δ_d	0.43907	$(0.28515, 0.61187)$	day^{-1}
$n_{\max}$	424.77	$(300.82, 1103.3)$	cell mm^{-2}
a	110.61	$(84.351, 151.43)$	cell mm^{-2}
b	0.35954	$(0.28151, 0.47076)$	dimensionless
